# Supramolecular Mille-Feuille: Adaptive Guest Inclusion in a New Aliphatic Guanidinium Monosulfonate Hydrogen-Bonded Framework

**DOI:** 10.1021/acs.cgd.4c00215

**Published:** 2024-04-04

**Authors:** Alexandra M. Dillon, Anna Yusov, Mohammad T. Chaudhry, Justin A. Newman, Krystyna M. Demkiw, K. A. Woerpel, Alfred Y. Lee, Michael D. Ward

**Affiliations:** † Department of Chemistry, New York University, New York, New York 10003, United States; ‡ Analytical Research and Development, Merck & Co., Inc., Rahway, New Jersey 07065, United States

## Abstract

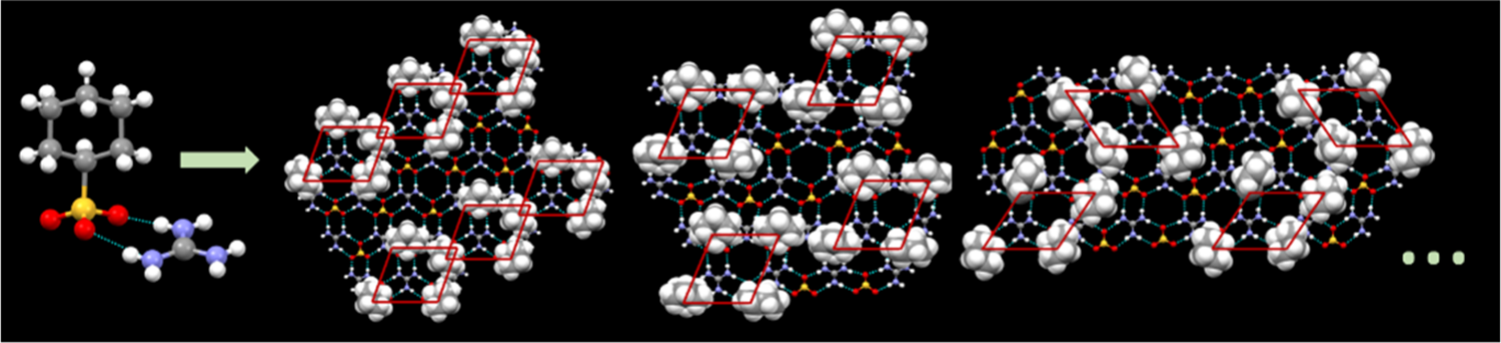

During the past three decades, the ability of guanidinium arenesulfonate host frameworks to encapsulate a wide range of guests has been amply demonstrated, with more than 700 inclusion compounds realized. Herein, we report crystalline inclusion compounds based on a new aliphatic host, guanidinium cyclohexanemonosulfonate, which surprisingly exhibits four heretofore unobserved architectures, as described by the projection topologies of the organosulfonate residues above and below hydrogen-bonded guanidinium sulfonate sheets. The inclusion compounds adopt a layer motif of guanidinium sulfonate sheets interleaved with guest molecules, resembling a mille-feuille pastry. The aliphatic character of this remarkably simple host, combined with access to greater architectural diversity and adaptability, enables the host framework to accommodate a wide range of guests and promises to expand the utility of guanidinium organosulfonate hosts.

## Abstract 2

A new hydrogen-bonded host−guanidinium cyclohexanemonosulfonate−forms new and unanticipated framework architectures that have not been observed previously in more than 700 reported guanidinium organosulfonate inclusion compounds, expanding the utility of these hosts for materials design and molecular structure determination of guest molecules.

## Introduction

In their classical 1984 Accounts of Chemical Research article “*Hydrogen-bond geometry in organic crystals*”,^1^ Taylor and Kennard presented statistics of hydrogen-bond angles culled from more than 1000 crystal structures. In that same year, they reported that charged donors and charged acceptors tended to form stronger hydrogen bonds than their corresponding uncharged donors and acceptors.^2^ This was a fertile time for advances in the role of hydrogen bonding in crystal packing,^3,4^ and Taylor and Kennard were among those who recognized that the investigation and use of hydrogen bonding for devising “rules” for rationalizing, or even predicting, crystal structures may be a “profitable area of research.” Notably, they also disclosed a comment by one of the reviewers of the Accounts article that “[rationalization of crystal structure hydrogen-bonding patterns]···is still at the foot of the rainbow.” Taylor and Kennard then stated: “The truth of this remark cannot be denied: we have come a long way, but there is much further to go.” Hydrogen bonding is now a staple of crystal design, and the impact of Kennard on advances in this area cannot be overstated.

**Figure 1:**
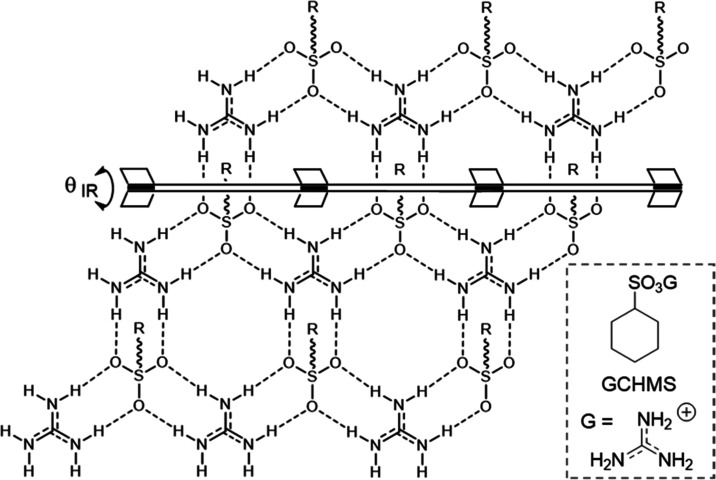
Quasi-hexagonal hydrogen-bonded sheet formed by guanidinium and organosulfonate ions. The sheet can be described as 1D GS “ribbons” fused along the ribbon edges by lateral (G)N^+^–H···^–^O(S) hydrogen bonds, which serve as flexible “hinges” that permit puckering of the sheet over a wide range of angles, denoted as the inter-ribbon puckering angle (θ_IR_) (Figure S1). The molecular structure of guanidinium cyclohexanemonosulfonate (GCHMS) is depicted at the bottom right.

Hydrogen-bonded frameworks based on guanidinium organosulfonates (GS), first reported in 1994, have emerged as a benchmark for crystal engineering, largely owing to a persistent “quasi-hexagonal” sheet-like network of guanidinium cations (G, C(NH_2_)_3_^+^) and organosulfonate anions (S, RSO_3_^–^) assembled through charge-assisted N–H···O hydrogen bonds (Figure 1).^5−7^ Database surveys performed by Taylor and Kennard^8^ were key to the analysis of hydrogen bonding in these compounds, and their insight regarding charge-assisted hydrogen bonding has been amply demonstrated. Organic residues projecting from the sulfonate nodes of the GS sheet provide a pathway for building in the third dimension, forming either guest-free phases by interdigitation of opposing residues or inclusion compounds when guest molecules are present during crystallization. The physicochemical environment of the inclusion cavities can be adjusted readily by changing the organosulfonate host. The size and shape of the cavities depend not only on the organosulfonate but also on the architecture of the framework, which in turn is influenced by the guest molecules that serve a templating role in inclusion compound formation.

GS host architectures can be classified by the projection topologies of the organic residues from either side of the GS sheet, which, in principle, permits an infinite number of framework isomers based on the disposition of the organosulfonate residues above and below the GS sheet.^7^ Different guests can template various framework architectures for a particular GS host and, conversely, different GS hosts can adopt different architectures for a particular guest, a phenomenon we coined “*architectural isomerism.*” This attribute enables GS hosts to form inclusion compounds with a wide range of guests, prompting their use for many applications.^9−11^ Furthermore, the GS sheet can pucker about a hydrogen-bond “hinge” (Figure 1) that joins adjacent hydrogen-bonded ribbons and permits the host framework to optimize host–guest packing. Collectively, these features contribute to the persistence of the GS sheet and the abundant number of GS inclusion compounds.

**Figure 2:**
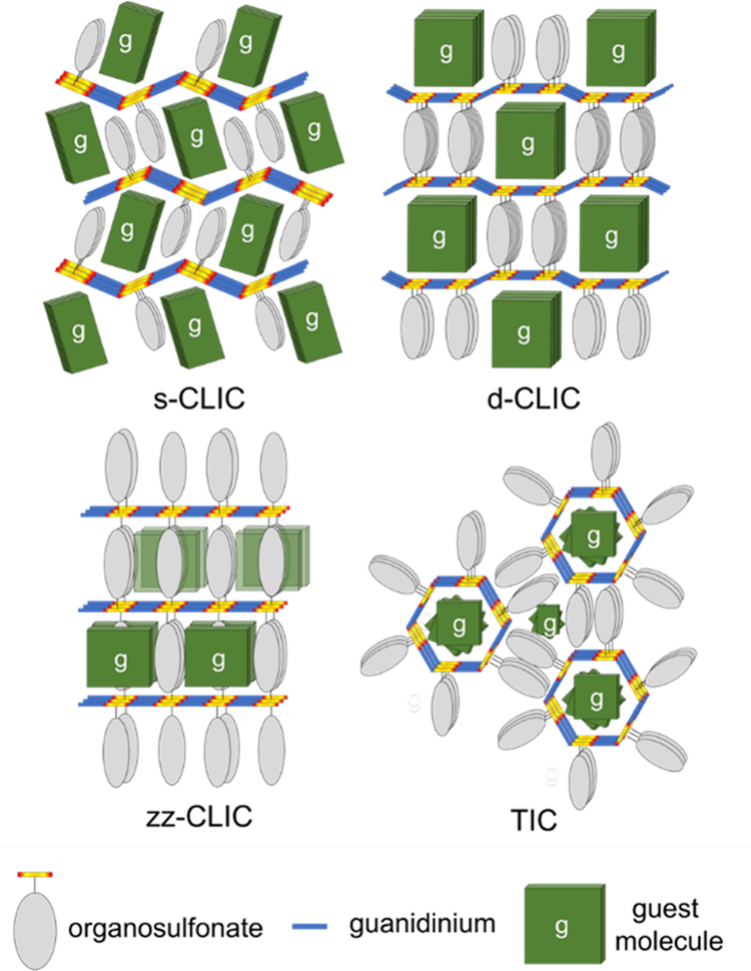
Schematic representations of the previously observed inclusion compound architectures for GS frameworks formed from guanidinium organomonosulfonate hosts. s-CLIC = simple continuous layered inclusion compound; d-CLIC = double continuous layered inclusion compound; zz-CLIC = zigzag continuous layered inclusion compound; and TIC = tubular inclusion compound. A single organomonosulfonate host can form each of these architectures as a consequence of guest templating, illustrating architectural isomerism. The zz-CLIC depiction here signifies two unique architectures formed by distinct projection topologies (zz-CLIC I and zz-CLIC II, Figure S2).

Guanidinium arenemonosulfonates readily form inclusion compounds as well as guest-free phases.^9,12,13^ A comprehensive study of 24 guanidinium arenemonosulfonate hosts and 26 small aromatic guests revealed the formation of more than 300 inclusion compounds, with molecular packing that suggested substantial π–π host–guest interactions.^14^ The hosts in these inclusion compounds exhibited five architectures described by their unique projection topologies (Figures 2 and S2). While guest-free phases have been reported for a limited number of aliphatic guanidinium monosulfonates,^6,15−20^ no inclusion compounds with aliphatic monosulfonates have been reported.

**Figure 3:**
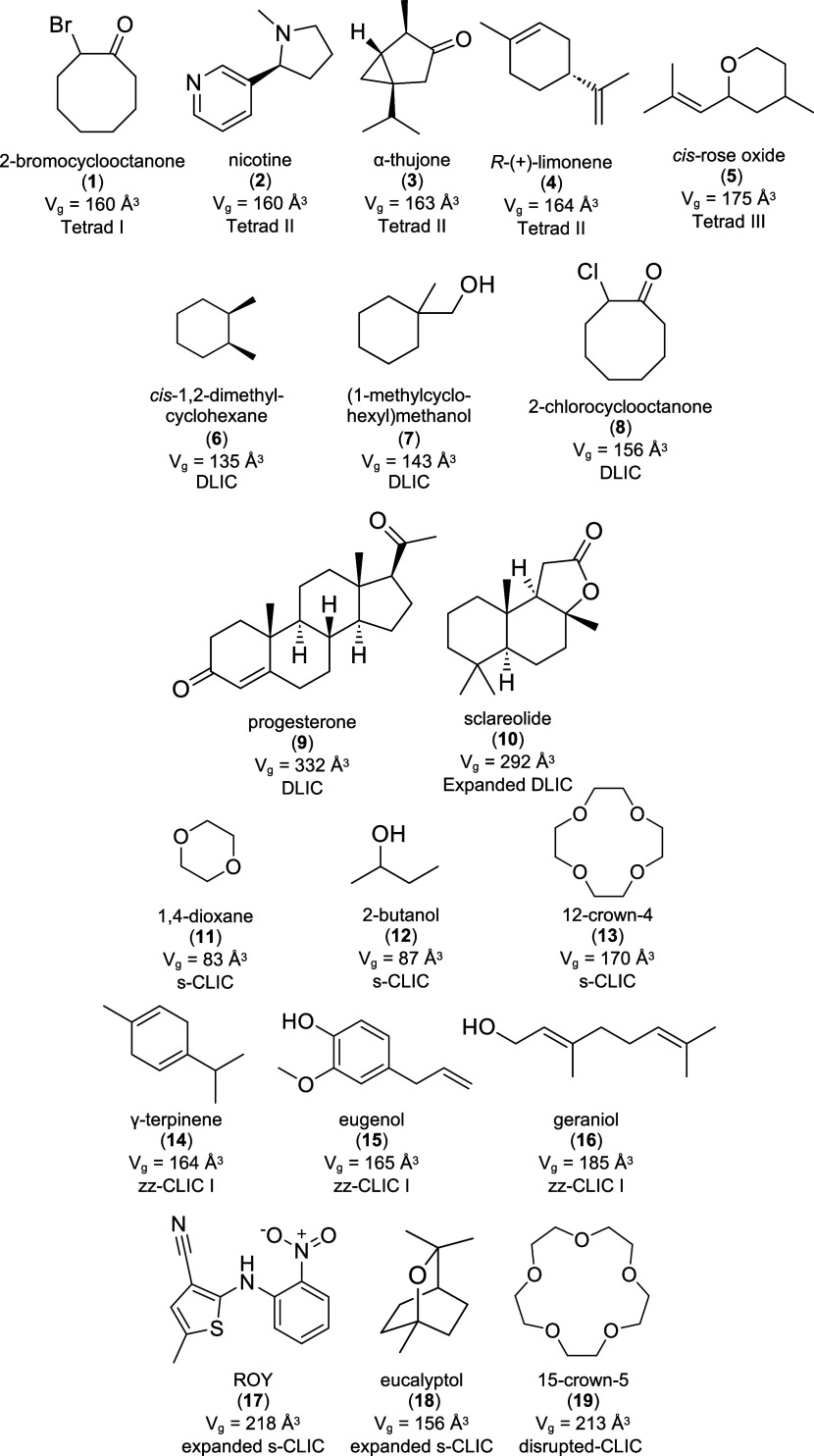
Molecular structures of the 19 guests included in the GCHMS host. Guest volumes (*V*_g_) and the corresponding framework architectures of the inclusion compounds are denoted below each guest structure. Compound **5** contains both stereoisomers of cis-rose oxide in equal amounts; the stereochemistry is not denoted here for the sake of clarity.

Recently, our laboratory deployed a new aliphatic host, guanidinium cyclohexanemonosulfonate (GCHMS). Herein, we report 19 new inclusion compounds based on the GCHMS host. The inclusion compounds adopt a layer motif of guanidinium sulfonate sheets interleaved with guest molecules, resembling a mille-feuille pastry. Despite more than 700 GS inclusion compounds reported over three decades, the GS host adopts four heretofore unobserved architectures, as described by the projection topologies of the organosulfonate residues above and below hydrogen-bonded guanidinium sulfonate sheets. This new host appears to be promiscuous with respect to guest inclusion, accommodating a wide range of guests with various shapes, sizes, and chemical characters, with guest volumes ranging from 83 to 332 Å^3^ (Figure 3) and host:guest stoichiometries from 1:1 to 6:1 for the 19 inclusion compounds. These architectures appear to exhibit a greater proclivity for the inclusion of aliphatic guests compared with guanidinium arenesulfonate hosts, increasing the versatility of the GS toolkit for the encapsulation of guest molecules.

## Results and Discussion

**Figure 4:**
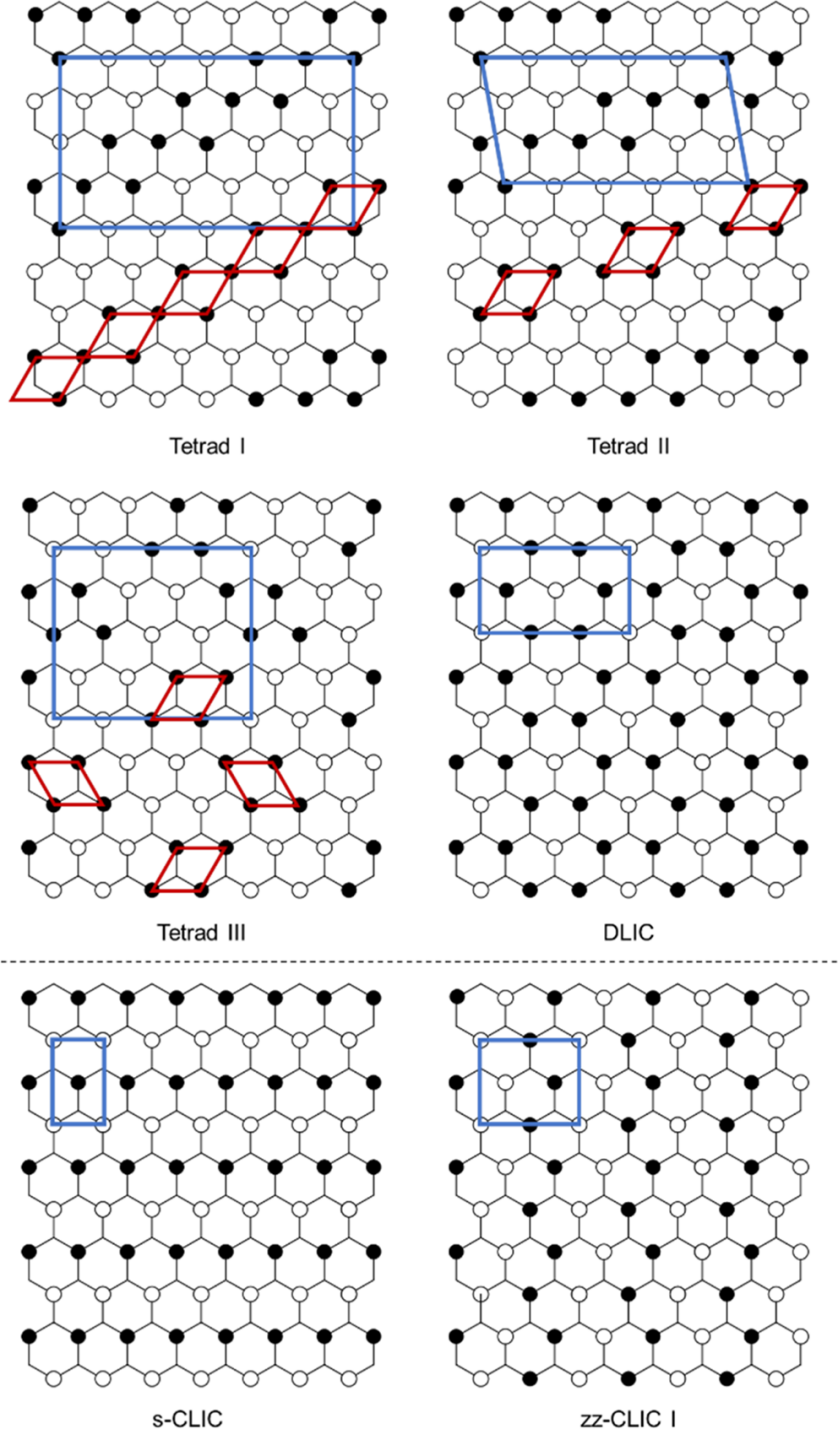
Projection topologies of the GS sheets observed in GCHMS inclusion compounds. The top four topologies have not been observed previously. Filled and open circles depict organic groups projecting from the sulfonate nodes above and below the sheet, respectively. The guanidinium ions sit on the undecorated nodes of the hexagonal tiling. The blue parallelograms depict the translational repeat unit of each sheet. The red parallelograms represent the “tetrad” repeat units that contain guest molecules in the Tetrad I–III architectures (Figure S4). The formalism that describes the unique projection sequence of that architecture can be found in Table S3.^7^

**Figure 5:**
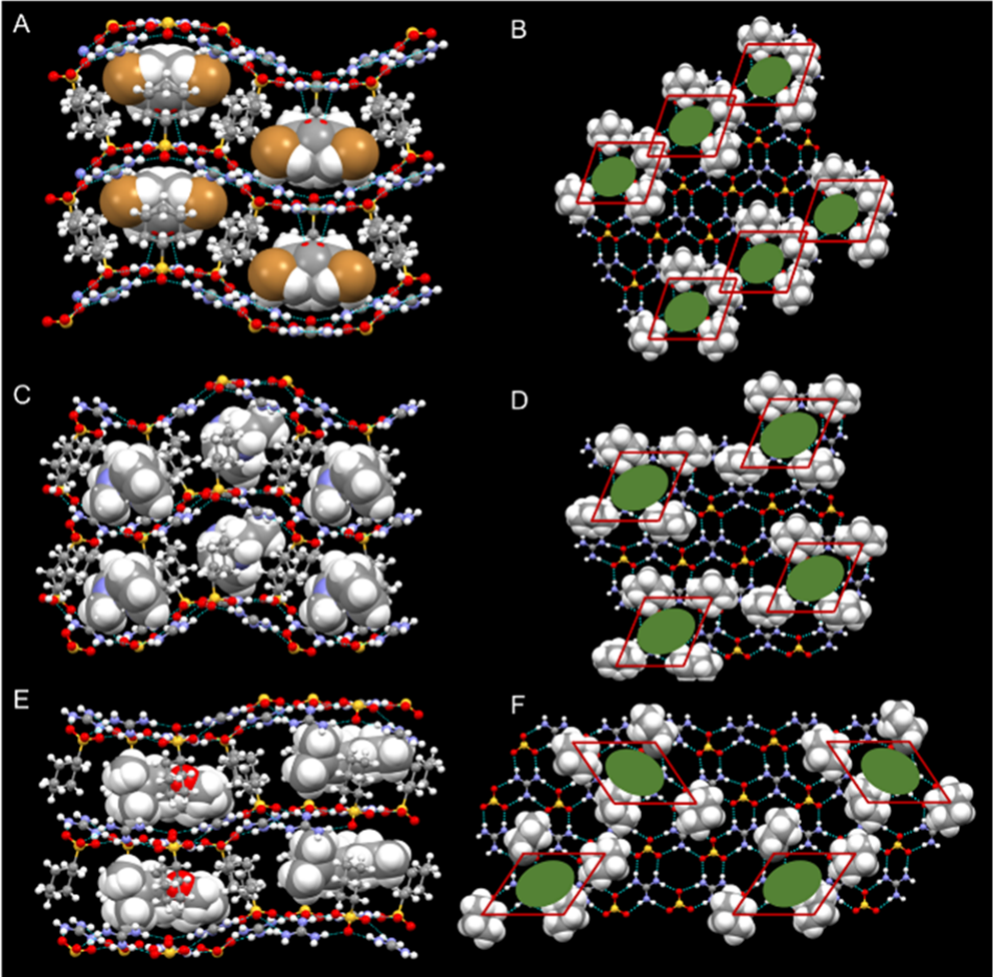
Crystal structures of (A, B) (GCHMS)_3_⊃2-bromocyclooctanone (**1**) in the Tetrad I architecture, (C, D) (GCHMS)_4_⊃nicotine (**2**) in the Tetrad II architecture and (E, F) (GCHMS)_4_⊃*cis*-rose oxide (**5**) in the Tetrad III architecture. The left panels depict the frameworks as ball-and-stick and the guest molecules as space-filling. Panels on the right illustrate top-down views of one side of each GS sheet with organic residues rendered as space-filling. Guest molecules are denoted as green ovals for the sake of clarity. Red parallelograms denote the repeat tetrad locations illustrated in Figure 4.

**Figure 6:**
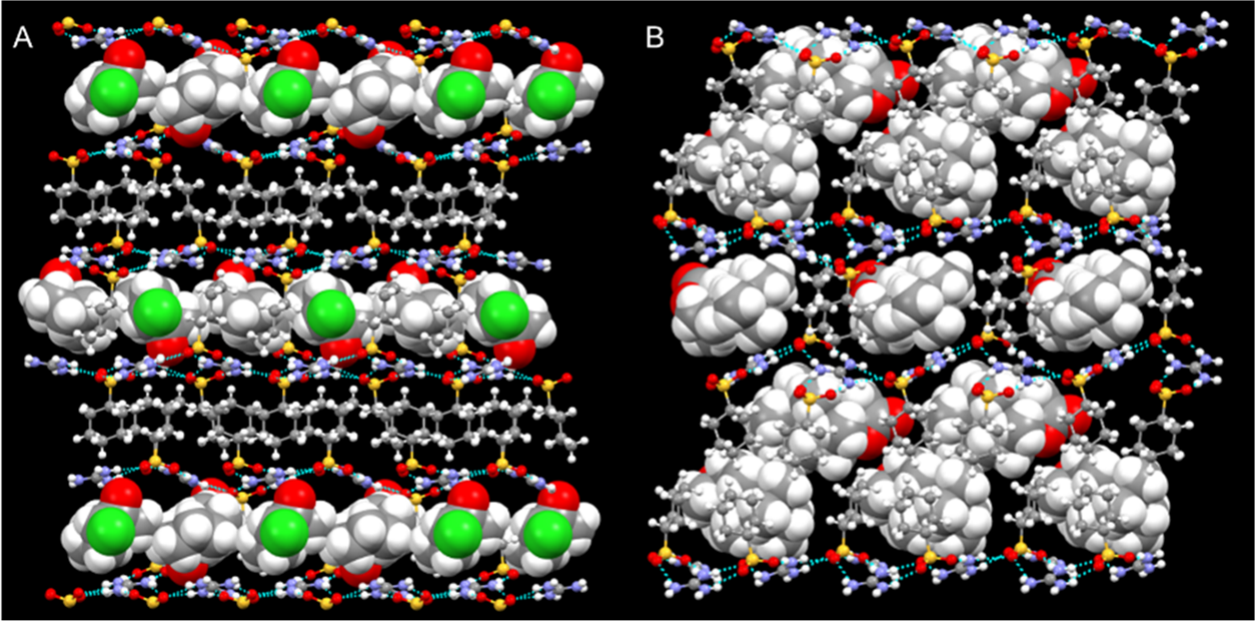
Crystal structure of (GCHMS)_3_⊃2-chlorocyclooctanone (**8**) in the DLIC architecture and (GCHMS)_2_⊃sclareolide (**10**) in an “expanded”-DLIC architecture. The frameworks are depicted as ball-and-stick and the guest molecules as space-filling.

The new GCHMS host architectures have unique “up–down” projection topologies that can be described graphically (Figure 4) or by a formalism based on their unique projection sequences (Table S3).^7^ The guest molecules serve as templates for these architectures, guiding the host assembly into a configuration that can accommodate the guest. Three new GCHMS architectures (Tetrad I–III) were observed wherein guest molecules sit within pockets formed by tetrads of cyclohexyl residues projecting from the same side of the hydrogen-bonded sheet. These architectures differ with respect to the positioning of tetrads relative to one another on the GS sheet (Figures 4 and S4). The CHMS residues are interdigitated with tetrads from the opposing sheet above. GCHMS crystallizes with 2-bromocyclooctanone in the Tetrad I architecture to afford inclusion compound (GCHMS)_3_⊃2-bromocyclooctanone (**1**) (Figure 5A). This guest was chosen to characterize its conformation with respect to stereoselective additions of nucleophilic reagents,^21,22^ the details of which will be described separately as part of a larger study. The projection topology is described by continuous rows of tetrads sharing a common node and separated by channels (Figures 4,5B and S4). The channels are occupied with identical rows of tetrads from the adjoining sheet. Each tetrad is occupied by a single guest molecule.

(GCHMS)_4_⊃nicotine (**2**) (Figure 5C), (GCHMS)_4_⊃α-thujone (**3**), and (GCHMS)_4_⊃(*R*)-(+)-limonene (**4**) inclusion compounds crystallize in the Tetrad II architecture, wherein the tetrads of CHMS are arranged in rows like Tetrad I but do not share a common node (Figures 4,5D and S4). The volumes of the guests in **2**–**4** (*V*_nicotine_ = 160 Å^3^; *V*_α-thujone_ = 162 Å^3^; *V*_(*R*)-(+)-limonene_ = 164 Å^3^) are nearly identical, which may explain their inclusion by the same host architecture despite drastically different shapes. Guest volume is not the sole factor, however, as **1** exhibits a different architecture despite having an identical volume (*V*_2-bromocyclooctanone_ = 160 Å^3^). (GCHMS)_4_⊃*cis*-rose oxide (**5**) adopts the Tetrad III architecture (Figure 5E), wherein each tetrad is related by a glide plane, resulting in a zigzag pattern of tetrads on the GS sheet (Figures 4,5F and S4). The larger host:guest ratios of 3:1 and 4:1 in the Tetrad I–III architectures have not been observed previously for GS compounds, which typically have host:guest stoichiometries less than or equal to one, indicating that GCHMS has the capacity to accommodate guests of larger sizes. The GS sheets in these inclusion compounds exhibit a wave-like puckering that reflects their well-demonstrated compliant character, enabling the host to accommodate the subtle steric demands of the guest.

(GCHMS)_3_⊃*cis*-1,2-dimethylcyclohexane (**6**), (GCHMS)_3_⊃(1-methylcyclohexyl)methanol (**7**), (GCHMS)_3_⊃2-chlorocyclooctanone (**8**), and (GCHMS)_6_⊃progesterone (**9**) adopt a new architecture, dubbed here as a discontinuous layered inclusion compound (DLIC) (Figure 6). The projection topology of organic residues on opposite sides of each GS sheet is inverted such that the number of organic residues projected from one side (A side) is twice that of the other (B side). The sheets are organized in alternating layers wherein each layer is composed of interdigitated residues from two A sides or two B sides. The lower density of cyclohexyl residues in the B side layer permits inclusion of guests, whereas the A side layer consists of densely packed, interdigitated CHMS residues. The host:guest ratios of inclusion compounds **6**, **7**, and **8** are 3:1 despite the differences in guest volumes (*V*_*cis*-1,2-dimethylcyclohexane_ = 135 Å^3^; *V*_(1-methylcyclohexyl)methanol_ = 143 Å^3^; *V*_2-chlorocyclooctanone_ = 156 Å^3^). The host:guest ratio of 6:1 in compound **9** reflects the large volume of progesterone guest (*V*_progesterone_ = 332 Å^3^), where the larger guest molecule resides in the space occupied by two guest molecules in compounds **6**–**8** (Figure S6).

(GCHMS)_2_⊃sclareolide (**10**) adopted an “expanded”-DLIC architecture, where the distance between opposing sheets in every other layer (alternating between *d*_sheet–sheet_*=* 7.717 and 15.435 Å) is substantially larger than that for all other inclusion compounds reported here (7.432 Å < *d*_sheet–sheet_ < 8.833 Å, Table S2) and for numerous inclusion compounds based on arenemonosulfonates.^14^ The expanded distances in every other layer are a consequence of guest inclusion that precludes the interdigitation of opposing CHMS residues projecting from the A side of the sheet. The location of the sclareolide guest molecules on the B side of the GS sheet is identical to that of compound **9** with progesterone guest, but the A side guest molecules occupy the location where the CHMS residues of the A side of the GS sheet would typically interdigitate, as observed in structures **6**–**9** (Figure S6). This results in a host:guest ratio of 2:1, unlike the other inclusion compounds in the DLIC architecture, and a structure wherein every other layer has double the number of guest molecules than their adjacent layers (Figure S6). The molecular volume of sclareolide (*V*_sclareolide_ = 292 Å^3^) rests between the smaller guests and progesterone guest that form the typical DLIC, demonstrating further the remarkable adaptability of the GCHMS host to adapt to the steric demands of a guest and achieve dense packing.

**Figure 7:**
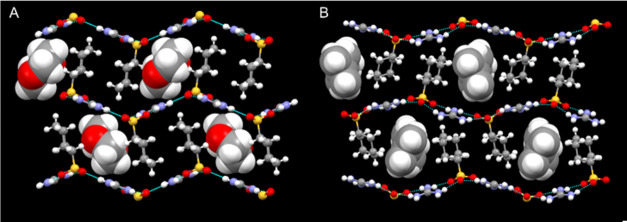
Crystal structures of (A) GCHMS⊃1,4-dioxane (**11**) in the s-CLIC architecture and (B) (GCHMS)_2_⊃γ-terpinene (**14**) in the zz-CLIC I architecture. The frameworks are depicted as ball-and-stick and the guest molecules as space-filling.

GCHMS also forms more typical lamellar architectures, including a guest-free phase in the s-CL architecture, identical to that reported previously for guanidinium monosulfonates (Figure S8 and Table S1).^14^ The topologically identical guest-containing s-CLIC architecture, reported for numerous guanidinium arenemonosulfonate inclusion compounds, was observed for (GCHMS)⊃1,4-dioxane (**11**, Figure 7A), (GCHMS)⊃2-butanol (**12**) and (GCHMS)_2_⊃12-crown-4 (**13**). The GS sheet in the guest-free phase of GCHMS is highly puckered (θ_IR_*=* 90°, Figure S8), once again revealing the compliance of the GS sheet that enables close-packing of the CHMS residues. Inclusion compounds **11**–**13**, however, exhibit larger θ_IR_ values (131, 140, and 136°, respectively) that accompany the creation of cavities that accommodate the guest molecules. The volumes of 2-butanol and 1,4-dioxane are nearly half that of 12-crown-4 (*V*_1,4-dioxane_ = 83 Å^3^; *V*_2-butanol_ = 87 Å^3^; *V*_12-crown-4_ = 170 Å^3^), which is reflected in the host:guest ratios of 1:1, 1:1, and 2:1, respectively.

(GCHMS)_2_⊃γ-terpinene (**14**, Figure 7B), (GCHMS)_2_⊃eugenol (**15**), and (GCHMS)_2_⊃geraniol (**16**) adopted the zz-CLIC I topology (Figure 4). The zz-CLIC I architecture accommodates guest molecules that are organized along the one-dimensional (1D) channels of the framework (*V*_γ-terpinene_ = 164 Å^3^; *V*_eugenol_ = 165 Å^3^; *V*_geraniol_ = 185 Å^3^) in a 2:1 host:guest ratio. Notably, (*R*)-(+)-limonene and γ-terpinene have identical molecular volumes (*V*_γ-terpinene_ = *V*_(*R*)-(+)-limonene_ = 164 Å^3^) but differ only with respect to the location of a double bond. Yet they prompted the formation of different architectures (tetrad II vs the zz-CLIC I, respectively). This example demonstrates that subtle differences in guest character can influence the host architecture.

**Figure 8:**
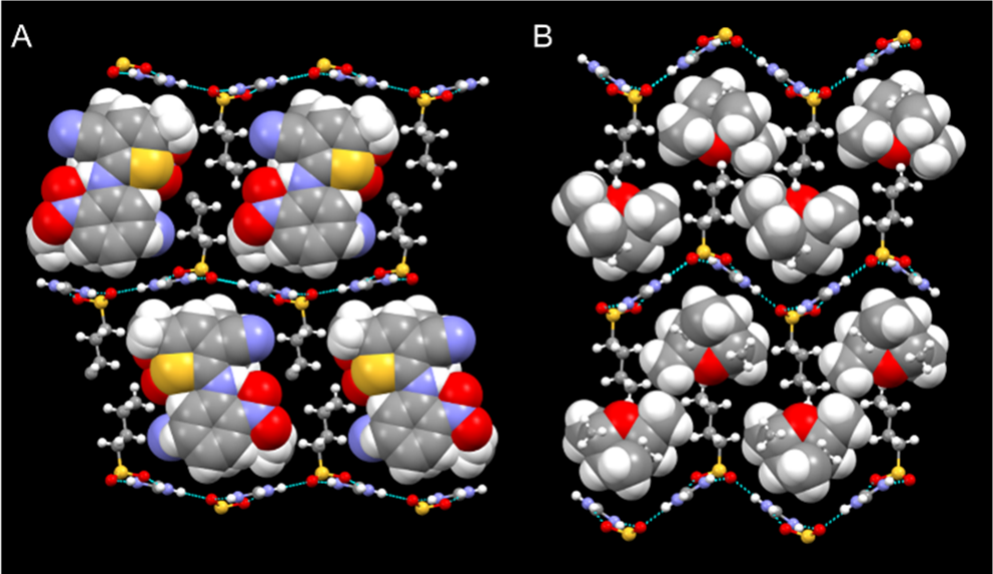
Crystal structures of (A) (GCHMS)_3_⊃ROY (**17**) and (B) (GCHMS)_3_⊃eucalyptol (**18**) in an s-CLIC architecture, where adjacent sheets are further apart than typically observed. The frameworks are depicted as ball-and-stick and the guest molecules as space-filling.

Eucalyptol and ROY (5-methyl-2-[(2-nitrophenyl)amino]-3-thiophenecarbonitrile), which is well-known for its conformational polymorphism,^23−26^ are included in the GCHMS host, both adopting the s-CLIC architecture with guests contained within 1D channels flanked by CHMS residues (Figure 8). The distances between adjacent GS sheets in GCHMS⊃ROY (**17**) and GCHMS⊃eucalyptol (**18**) (*d*_sheet–sheet_*=* 13.01 and 13.65 Å, respectively) are substantially larger than those for all other inclusion compounds reported here (7.432 Å < *d*_sheet–sheet_ < 8.833 Å, Table S2), except for (GCHMS)_2_⊃sclareolide (**10)**. These expanded distances are again a consequence of guest inclusion, which precludes interdigitation of opposing CHMS residues. A similar example of an expanded s-CLIC architecture was reported recently for the inclusion compound formed from guanidinium 1-naphthalenesulfonate and tetracyanoquinodimethane.^27^ The GS sheets in **17** and **18** are puckered (θ_IR_*=* 152 and 118°, respectively), which allows the host to “shrink-wrap” and achieve dense packing between the CHMS residues and the guest molecules. In **17**, CHMS residues projecting from opposing sheets are eclipsed, resulting in channels with 1D stacks of ROY molecules (Figure 8A). The host residues in **18**, however, are offset from one another, effectively doubling the number of 1D channels to accommodate the smaller guest (*V*_ROY_ = 218 Å^3^ vs *V*_eucalyptol_ = 156 Å^3^, Figure 8B). Although the volumes of eucalyptol and 2-chlorocyclooctanone (inclusion compound **8**) are identical (*V*_eucalyptol_ = *V*_2-chlorocyclooctanone_ = 156 Å^3^), their inclusion compounds result in different architectures.

As previously demonstrated in other GS systems, the GCHMS host framework can remain intact despite competitive hydrogen bonding that disrupts the ideal quasi-hexagonal GS sheet. GCHMS⊃15-crown-5 (**19**) formed a s-CLIC architecture with a disrupted GS sheet that accommodates the guest in a 1:1 host:guest ratio despite the large volume of the guest (*V*_15-crown-5_ = 213 Å^3^). Hydrogen bonds between the guanidinium protons and two oxygen atoms of the 15-crown-5 guest disrupt the sheet to produce isolated hydrogen-bonded ribbons (Figure S9). Though the framework of **19** lacks the typical GS sheet motif, its structure demonstrates the tolerance of the GS host for guests with hydrogen-bonding character.

## Conclusions

The GCHMS inclusion compounds described here confirm once again that GS host architectures are a consequence of the size, shape, and character of the host and guest. The numerous host architectures, including four new ones, further demonstrate the important templating role of guest molecules. Moreover, GCHMS readily forms inclusion compounds despite the absence of cavities that are preordained in polyvalent sulfonates. The lack of a covalent connection between GS sheets, in contrast to guanidinium di- and polysulfonates, removes the constraint for registry between opposing sheets, enabling new unique architectures and the inclusion of a wide range of guest molecules. Furthermore, GCHMS appears to be distinct from other hosts in its ability to trap larger molecules with greater host:guest ratios. The aliphatic character of the GCHMS host may prove favorable for inclusion of aliphatic guests compared with guanidinium arenesulfonates, which have structures that appear to be governed by π–π host–guest and host–host interactions. In the absence of π–π interactions, it is reasonable to suggest that favorable entropic effects associated with desolvation of the GCHMS residues and guest molecules play an even more important role in inclusion compound formation, contributing to the apparent promiscuity of this host. The aliphatic character of guanidinium cyclohexanemonosulfonate host, combined with access to even more architectures beyond those reported previously, promises to expand the utility of guanidinium organosulfonate hosts, whether for design of functional materials^28−33^ or molecular structure determination of encapsulated guests.

## Supporting Information Available

Crystallographic data; X-ray characterization details; experimental procedures; X-ray tables; previously reported projection topologies; projection formalisms; the guest-free structure of GCHMS; projection topologies with symbols for guest locations; and additional figures of architecture types (PDF)
