# Supplementary material for: Supramolecular Mille-Feuille: Adaptive Guest Inclusion in a New Aliphatic Guanidinium Monosulfonate Hydrogen-Bonded Framework
Source: Cryst Growth Des. 2024 Apr 4;24(8):3483–90. doi: 10.1021/acs.cgd.4c00215 (PMC11036357; doi:10.1021/acs.cgd.4c00215)
Supplement: Supplementary file 1 — cg4c00215_si_001.pdf [file cg4c00215_si_001.pdf]

Supramolecular Mille-feuille: Adaptive guest inclusion in a new aliphatic guanidinium monosulfonate hydrogen-bonded framework

Alexandra M. Dillon[a], Anna Yusov[a], Mohammad T. Chaudhry[b], Justin A. Newman[b], Krystyna M. Demkiw[a], K. A. Woerpel[a], Alfred Y. Lee[b*], and Michael D. Ward[a*]

[a]*Department of Chemistry, New York University, New York City, New York, 10003, United States. *Email: mdw3@nyu.edu*

[b]*Analytical Research and Development, Merck & Co., Inc., Rahway, New Jersey, 07065, United States. *Email: alfred.lee@merck.com*

Supplementary information

Contents

Materials and Methods.

Table S1. Detailed crystallographic data of inclusion compounds **1-19**, and guest-free GCHMS (**20**).

Table S2. Details of the GCHMS inclusion compounds **1-19**, and guest-free GCHMS (**20**), with guest, guest volume, host:guest ratios, crystalline architecture, puckering angle (where applicable), and sheet-to-sheet distances.

Figure S1. Inter-ribbon puckering angle (θ_{IR}) in (GCHMS)⊃1,4-dioxane (**11**).

Figure S2. Previously reported projection topologies of guanidinium monosulfonate inclusion compounds.

Table S3. Projection formalism for each monosulfonate architecture type.

Figure S3. Projection topologies of GCHMS architectures with symbols to depict guest location relative to the hydrogen-bonded sheet.

Figure S4. Projection topologies of Tetrad I-III architectures with parallelograms to depict tetrad location on the hydrogen-bonded sheet.

Figure S5. Top-down views of the crystal structure of one side of the GS sheet for each Tetrad architecture type, represented by inclusion compounds **1**, **2** and **5**.

Figure S6. Top-down views of the crystal structure of both sides (A and B) of the GS sheet in **8** and **9**, representing the DLIC architecture, and **10** in the "expanded"-DLIC architecture.

Figure S7. Top-down view of the crystal structures of one side of the GS sheets in (A) **11** and (B) **14**, representing the CLIC and zz-CLIC architectures, respectively.

Figure S8. The crystal structure of the guest-free phase of GCHMS (**20**) in the s-CL architecture.

Figure S9. The one-dimensional hydrogen bonded ribbons in **19**.

References.

Materials

Cyclohexane sulfonic acid was purchased from Sigma-Aldrich (St. Louis, MO). Guests 2-butanol, dioxane, *cis*-1,2-dimethylcyclohexane, *R*-(+)-limonene, α-thujone, and 15-crown-5 were purchased from Sigma Aldrich. Guests geraniol, eugenol, and nicotine were purchased from VWR (Randor, PA). 12-crown-4 was purchased from Aaron Chemicals (San Diego, CA). *cis*-Rose oxide and eucalyptol were purchased from 1PlusChem. Gamma-terpinene was purchased from A2B Chem (San Diego, CA). 5-Methyl-2-[(2-nitrophenyl)amino]-3-thiophenecarbonitrile was purchased from Ambeed (Arlington Heights, IL). All reagents were used with no additional purification. Guests 2-bromocyclooctanone and 2-chlorocyclooctanone were synthesized according to previously reported procedures.[1]

The apo-host salt, guanidium cyclohexanesulfonate, was prepared by combining acetone solutions of approximately 1.10 molar equivalents of guanidinium tetrafluoroborate and 1.0 molar equivalent of a the organosulfonic acid to produce a precipitate of the corresponding guest-free apo-host. The mixture was dried in a rotary evaporator and the resulting solid was washed with acetone several times and dried in vacuo affording the apo-host salt of guanidium cyclohexanesulfonate.

Methods

Characterization methods. Crystals were mounted on a MiTeGen MicroMount with Type B immersion oil (Cargille Labs). Single crystal X-ray diffraction data was obtained using several instruments. Single crystal x-ray diffraction data for compounds **7** and **9-13** were collected at 100 K on a Bruker SMART APEX II diffractometer equipped with a PHOTON-II-C14 detector. The X-ray beam generated from an INCOTEC micro-focused Mo source was monochromated and collimated by a Montel multilayer optics. The wavelength from the Mo Kα radiation is 0.71073 Å. Crystal temperature was controlled by an Oxford Cryosystems 700+ Cooler. For each crystal, a phi scan (APEX4) was performed to evaluate the crystal quality and determine the data collection parameters. Full datasets were collected with omega scan methods. The data sets were processed with the INTEGRATE program of the APEX4 software for reduction and cell refinement.[2] Multi-scan absorption corrections were applied by the SCALE program for the area detector. Single crystal x-ray diffraction data for compounds **1**, **6**, **8**, **19** and the guest-free structure of GCHMS (**20**) were collected at 100 K using a Rigaku XtaLAB synergy instrument equipped with graphite monochromated copper Kα (λ = 1.5406 Å) radiation and a HyPix-6000HE detector. Unless otherwise specified, data were collected to a resolution of 0.837 Å, as per IUCr guidelines. Data was processed using CrysAlisPro V 1.171.42.54A, and absorption corrections were done using spherical harmonics or through face indexing using SCALE3 ABSPACK. Single crystal x-ray diffraction data for compound **2** was collected at 100 K on a Rigaku XtaLAB Synergy R (DW system) equipped with a HyPix-Arc 100 Hybrid Pixel Array Detector. The X-ray beam was generated by VariMax DW dual-wavelength confocal optics. Sample temperature was maintained at 100 K with the Oxford Cobra open-flow cooler, and samples were mounted on nylon loops from Hampton Research. Single crystal x-ray diffraction data for compounds **3**, **4**, **5** and **14-18** were collected at 130 K on a Bruker D8 VENTURE diffractometer using Cu Kα radiation. The structures were solved by intrinsic phasing methods (SHEL-XT or SHEL-XS) and the structure models were completed and refined using the full-matrix least-square methods on F2 (SHELXL).[3,4] Non-hydrogen atoms in the structures were refined with anisotropic displacement parameters, and hydrogen atoms on carbons were placed in idealized positions (C-H = 0.95-1.00 Å) and included as riding with Uiso(H) = 1.2 or 1.5 Ueq(non-H). OLEX2 solvent mask was used to omit heavily disordered guest molecules that could not be refined in compound **15**.[5] Graphical representation of crystallographic data was done using Mercury

(Version 4.3.1).[6] Crystallographic data of these structures, including cif, res, fcf, and hkl files, have been deposited with the Cambridge Crystallographic Data Centre with Numbers **2307223** (for **1**), **2307209** (for **2**), **2307213** (for **3**), **2308623** (for **4**), **2307217** (for **5**), **2307207** (for **6**), **2307210** (for **7**), **2307222** (for **8**), **2326859** (for **9**), **2326858** (for **10**), **2307211** (for **11**), **2307206** (for **12**), **2307215** (for **13**), **2307214** (for **14**), **2307216** (for **15**), **2307218** (for **16**), **2307193** (for **17**), **2307212** (for **18**), **2307196** (for **19**), and **2307220** (for guest-free GCHMS, **20**). Copies of these data can be requested, free of charge, from the CCDC website at https://www.ccdc.cam.ac.uk/structures/.

Crystallization methods. Single crystals of inclusion compounds were obtained by evaporation of a 2:1 solution of methanol:ethanol (1 mL) containing the guanidinium organosulfonate (GS) apohost (5 mM) and the target guest molecule (10 mg) under ambient temperature over several days.

Single crystals of **17** were obtained by slow evaporation of a 1:1 solution of methanol:acetonitrile containing 5-methyl-2-[(2-nitrophenyl)amino]-3-thiophenecarbonitrile (ROY) (1.3 mg in 500 μL of acetonitrile) and the GCHMS apohost (1.1 mg in 500 μL of methanol) under ambient temperature over several days.

Volume calculations. Molecular volumes were calculated by the formula published by Zhao et al.[7]

Table S1. Detailed crystallographic data.

**Compound name**	(GCHMS)$_3$⊂2-bromocyclooctanone	(GCHMS)$_4$⊃ nicotine	(GCHMS)$_4$⊃α-thujone	(GCHMS)$_4$⊃ (*R*)-(+)-limonene
**Structure file name**	**1**	**2**	**3**	**4**
**X-ray lab code**	mds056	23mdw80ay	23mdw69ay	AY129F
**CCDC no.**	2307223	2307209	2307213	2308623
**Formula by X-ray**	$C_{29}H_{64}BrN_9O_{10}S_3$	$C_{38}H_{82}N_{14}O_{12}S_4$	$C_{66}H_{152}N_{24}O_{25}S_8$	$C_{36.49}H_{81.58}N_{12}O_{12}S_4$
**Formula weight**	875.0	1055.4	1938.59	1008.77
**Crystal habit**	Colorless plate	Colorless plate	Clear colorless plate	Colorless plate
**Crystal size (mm)**	0.230 x 0.150 x 0.020	0.360 x 0.220 x 0.020	0.22 x 0.12 x 0.01	0.220 x 0.170 x 0.020
**Crystal system**	Orthorhombic	Triclinic	Triclinic	Triclinic
**Space group (no.)**	*Pmn2₁* (31)	*P1* (1)	*P1* (1)	*P1* (1)
***a* (Å)**	20.6941(5)	8.58540(10)	8.6135(6)	8.5832(4)
***b* (Å)**	8.4766(3)	17.5870(2)	17.5833(12)	17.6798(7)
***c* (Å)**	12.3109(3)	19.2061(3)	19.1134(12)	19.0779(7)
α **(°)**	90	98.9960(10)	80.851(5)	81.553(3)
β **(°)**	90	90.0870(10)	89.256(5)	89.200(3)
γ **(°)**	90	91.6470(10)	89.920(5)	89.848(3)
***V* (Å^3)**	2159.52(11)	2863.05(7)	2857.7(3)	2863.4(2)
Z	2	2	1	2
**D_c (g cm^{-3})**	1.346	1.224	1.126	1.170
***F*(000)**	928.0	1136	1044	1089
**μ (mm^{-1})**	3.145	2.055	2.014	2.021
**Total reflections**	10457	55115	100701	153676
**Unique reflections**	3394	19286	19437	22086
**R_{int}**	0.0374	0.0237	0.1010	0.0694
**R_1[a] [$I > 2\sigma(I)$]**	0.0628	0.0271	0.1382	0.0644
**wR_2[b] (all data)**	0.1765	0.0746	0.3514	0.1798
**GOF (all data)**	1.044	1.066	1.125	1.042
**Flack parameter[c]**	-0.006(19)	0.020(3)	-0.05(4)	-0.062(13)

[a]$R_1 = \Sigma||F_o|-|F_c||/\Sigma|F_o|$. [b]$wR_2=\{\Sigma[w(F_o^2-F_c^2)^2]/\Sigma[w(F_o^2)^2]\}^{1/2}$. [c]N/A – not applicable.

Table S1 (continued). Detailed crystallographic data.

**Compound name**	(GCHMS)$_4$⊂*cis*-rose oxide	(GCHMS)$_3$⊃*cis*-1,2-dimethylcyclohexane	(GCHMS)$_3$⊃(1-methyl cyclohexyl)methanol	(GCHMS)$_3$⊃2-chlorocyclooctanone
**Structure file name**	**5**	**6**	**7**	**8**
**X-ray lab code**	23mdw67ay	Mds058	23mdw19d	mdr054
**CCDC no.**	2307217	2307207	2307210	2307222
**Formula by X-ray**	$C_{38}H_{84}N_{12}O_{13}S_4$	$C_{29}H_{67}N_9O_9S_3$	$C_{29}H_{67}N_9O_{10}S_3$	$C_{25.97}H_{59.07}Cl_{0.62}N_9O_{9.62}S_3$
**Formula weight**	1045.41	782.09	798.09	833.54
**Crystal habit**	Clear colorless plate	Colorless needle	Colorless needle	Colorless plate
**Crystal size (mm)**	0.510 x 0.120 x 0.020	0.560 x 0.090 x 0.030	0.330 x 0.090 x 0.060	0.490 x 0.090 x 0.040
**Crystal system**	Orthorhombic	Orthorhombic	Monoclinic	Monoclinic
**Space group (no.)**	$Pca2_1$ (29)	*Cmca* (64)	$P\ 2_1\ /\ c$ (14)	$P\ 2_1\ /\ c$ (14)
a **(Å)**	28.4245(6)	21.6164(6)	12.4616(6)	12.4187(2)
b **(Å)**	8.4862(2)	12.4875(4)	31.3075(14)	31.3315(4)
c **(Å)**	23.8945(5)	31.7055(10)	12.3952(6)	12.52302(19)
**α (°)**	90	90	90	90
**β (°)**	90	90	119.285(2)	119.397(4)
**γ (°)**	90	90	90	90
**V (Å^3)**	5763.7(2)	8558.4(5)	4217.8(4)	4245.27(14)
Z	4	8	4	4
**D_c (g cm^{-3})**	1.205	1.214	1.257	1.204
***F*(000)**	2256	3392	1728	1654
**μ (mm^{-1})**	0.064	2.043	0.234	2.415
**Total reflections**	125128	18321	75764	52511
**Unique reflections**	11725	4292	10533	8183
**R_{int}**	0.0777	0.0514	0.0745	0.0428
**R_1^a [$I > 2\sigma(I)$]**	0.0679	0.0772	0.0636	0.0747
**wR_2^b (all data)**	0.1736	0.2000	0.1629	0.2400
**GOF (all data)**	1.043	1.131	1.064	1.038
**Flack parameter[c]**	-0.01(2)	n/a	n/a	n/a

[a]$R_1 = \Sigma||F_o|-|F_c||/\Sigma|F_o|$. [b]$wR_2=\{\Sigma[w(F_o^2-F_c^2)^2]/\Sigma[w(F_o^2)^2]\}^{1/2}$. [c]N/A – not applicable.

Table S1 (continued). Detailed crystallographic data.

**Compound name**	$(GCHMS)_6\supset$ progesterone	$(GCHMS)_6\supset$ sclareolide	$GCHMS\supset$ 1,4-dioxane	$GCHMS\supset$2-butanol
**Structure file name**	**9**	**10**	**11**	**12**
**X-ray lab code**	23mdw153d	24mdw1d	23mdw22d	23mdw57ay
**CCDC no.**	2326859	2326858	2307211	2307206
**Formula by X-ray**	$C_{63}H_{132}N_{18}O_{20}S_6$	$C_{90}H_{180}N_{18}O_{24}S_6$	$C_{18}H_{42}N_6O_8S_2$	$C_{18}H_{44}N_6O_7$S2
**Formula weight**	1654.22	2090.87	534.69	520.71
**Crystal habit**	Clear colorless block	Clear colorless plate	Clear colorless block	Clear colorless plate
**Crystal size (mm)**	0.479 x 0.230 x 0.064	0.150 x 0.100 x 0.060	0.54 x 0.33 x 0.03	0.480 x 0.410 x 0.060
**Crystal system**	Triclinic	Triclinic	Monoclinic	Monoclinic
**Space group (no.)**	*P1* (1)	*P1* (1)	$P\ 2_1/n$ (14)	$P\ 2_1/n$ (14)
***a* (Å)**	12.139(2)	11.8609(15)	7.1139(16)	7.1238(5)
***b* (Å)**	12.389(3)	12.1137(16)	15.034(3)	14.8642(10)
***c* (Å)**	17.674(3)	23.153(3)	12.418(3)	12.7990(9)
α **(°)**	79.048(15)	91.996(8)	90	90
β **(°)**	71.303(10)	102.195(8)	91.364(12)	93.933(4)
γ **(°)**	61.113(9)	118.607(7)	90	90
V **(Å^3)**	2202.5(8)	2818.5(7)	1327.8(5)	1352.09(16)
Z	1	1	2	2
D_c **(g cm^{-3})**	1.247	1.232	1.337	1.279
***F*(000)**	892	1134	576	564
μ **(mm^{-1})**	0.227	0.194	2.71	0.243
**Total reflections**	25822	34770	6331	9903
**Unique reflections**	11874	15261	2928	5434
R_{int}[d]	0.0886	0.0748	n/a	0.0558
R_1[a] $[I > 2\sigma(I)]$	0.0691	0.0663	0.0947	0.0630
wR_2[b] **(all data)**	0.1460	0.1604	0.2590	0.1392
**GOF (all data)**	1.066	1.039	1.189	1.125
**Flack parameter**[c]	0.04(7)	0.09(5)	n/a	n/a

[a]$R_1 = \Sigma||F_o|-|F_c||/\Sigma|F_o|$. [b]$wR_2=\{\Sigma[w(F_o^2-F_c^2)^2]/\Sigma[w(F_o^2)^2]\}^{1/2}$. [c]N/A – not applicable. [d]N/A – not applicable because the structure is twinned.

Table S1 (continued). Detailed crystallographic data.

**Compound name**	(GCHMS)$_2$⊃ 12-crown-4	(GCHMS)$_2$⊃ γ-terpinene	(GCHMS)$_2$⊃ eugenol [d]	(GCHMS)$_2$⊃ geraniol [e]
**Structure file name**	**13**	**14**	**15**	**16**
**X-ray lab code**	23mdw93ay	ay138	79ay	AY129-G
**CCDC no.**	2307215	2307214	2307216	2307218
**Formula by X-ray**	$C_{22}H_{50}N_6O_{10}S_2$	$C_{38}H_{84}N_{12}O_{12}S_4$	$C_{14}H_{34}N_6O_{6.5}S_2$	$C_{19}H_{43}N_6O_{6.5}S_2$
**Formula weight**	622.80	1029.41	446.59	523.71
**Crystal habit**	Clear colorless hexagon	Clear colorless needle	Clear colorless block	Colorless plate
**Crystal size (mm)**	0.42 x 0.33 x 0.16	0.46 x 0.02 x 0.01	0.31 x 0.05 x 2	0.36 x 0.19 x 0.02
**Crystal system**	Monoclinic	Monoclinic	Monoclinic	Monoclinic
**Space group (no.)**	$P\ 2_1\ /\ n$ (14)	*Pc* (7)	Cc (9)	$P\ 2_1\ /\ c$ (14)
***a* (Å)**	7.1579(14)	12.1982(5)	21.3686(15)	12.3748(6)
***b* (Å)**	17.353(4)	14.1654(6)	13.8975(10)	15.9156(7)
***c* (Å)**	12.466(3)	16.5069(7)	12.2639(8)	14.4666(7)
**α (°)**	90	90	90	90
**β (°)**	90.084(6)	90.010(3)	124.914(4)	90.821(2)
**γ (°)**	90	90	90	90
**V (Å^3)**	1548.4(5)	2852.3(2)	2986.5(4)	2848.9(2)
Z	2	2	4	4
**D_c (g cm^{-3})**	1.336	1.199	0.993	1.221
***F*(000)**	672	1112	960	1132
**μ (mm^{-1})**	0.231	2.039	1.886	2.063
**Total reflections**	28321	44919	27675	61315
**Unique reflections**	3872	9026	4719	5137
**R_{int}**	0.0617	0.1431	0.0883	0.0686
**R_1[a] [$I > 2\sigma(I)$]**	0.0611	0.1453	0.0655	0.0454
**wR_2[b] (all data)**	0.1521	0.3134	0.1830	0.1298
**GOF (all data)**	1.136	1.256	1.142	1.043
**Flack parameter[c]**	n/a	0.135(18)	0.38(4)	n/a

[a]$R_1 = \Sigma||F_o|-|F_c||/\Sigma|F_o|$. [b]$wR_2=\{\Sigma[w(F_o^2-F_c^2)^2]/\Sigma[w(F_o^2)^2]\}^{1/2}$. [c]N/A – not applicable. [d]Molecular formula reflects a solvent masked structure wherein the eugenol has been removed. [e]The geraniol guest is disordered about an inversion center in the void, therefore the refinement used a model wherein one of the 50% occupancy guests was removed, reflected in the molecular formula.

Table S1 (continued). Detailed crystallographic data.

**Compound name**	GCHMS⊃ROY	GCHMS⊃eucalyptol	GCHMS⊂15-crown-5	GCHMS guest-free
**Structure file name**	**17**	**18**	**19**	**20**
**X-ray lab code**	23mdw35d	23mdw110ay	Mds190	36d<br>mds186
**CCDC no.**	2307193	2307212	2307196	2307220
**Formula by X-ray**	$C_{19}H_{26}N_6O_5S_2$	$C_{17}H_{35}N_3O_4S$	$C_{34}H_{74}N_6O_{16}S_2$	$C_7H_{17}N_3O_3S_1$
**Formula weight**	482.58	377.54	887.11	223.29
**Crystal habit**	Orange plate	Clear colorless plate	Clear colorless plate	Clear colorless block
**Crystal size (mm)**	0.58 x 0.16 x 0.02	0.44 x 0.12 x 0.01	0.367 x 0.169 x 0.085	0.484 x 0.126 x 0.090
**Crystal system**	Orthorhombic	Monoclinic	Monoclinic	Orthorhombic
**Space group (no.)**	*Pnma* (62)	$P\ 2_1/n$ (14)	$P\ 2_1/c$ (14)	*Pnma* (62)
***a* (Å)**	26.0394(12)	7.0137(7)	19.9930(2)	16.8722(5)
***b* (Å)**	6.9906(3)	11.1893(12)	15.8102(2)	7.4485(2)
***c* (Å)**	12.8066(6)	27.288(3)	14.62810(10)	9.3238(3)
α **(°)**	90	90	90	90
β **(°)**	90	90.252(7)	97.0360(10)	90
γ **(°)**	90	90	90	90
V **(Å³)**	2331.20(18)	2141.5(4)	4589.02(8)	1171.75(6)
Z	4	4	4	4
D_c **(g cm⁻³)**	1.375	1.171	1.284	1.266
***F*(000)**	1016	824	1920	480
μ **(mm⁻¹)**	2.440	1.542	1.652	2.404
**Total reflections**	37002	25647	43902	6035
**Unique reflections**	2568	3282	9103	1207
R_{int}	0.0743	0.0885	0.0357	0.0328
R_1[a] $[I > 2\sigma(I)]$	0.1133	0.1135	0.0559	0.0325
wR_2[b] **(all data)**	0.2431	0.2690	0.1560	0.0876
**GOF (all data)**	1.416	1.230	1.029	1.089
**Flack parameter**[c]	n/a	n/a	n/a	n/a

[a] $R_1 = \Sigma||F_o|-|F_c||/\Sigma|F_o|$. [b] $wR_2=\{\Sigma[w(F_o^2-F_c^2)^2]/\Sigma[w(F_o^2)^2]\}^{1/2}$. [c] N/A – not applicable.

Table S2. Structural features of the GCHMS inclusion compounds **1-19** and the guest-free GCHMS (**20**).

Inclusion compound #	Guest	Guest volume (Å^3)	Host: guest ratio	Architecture	θ_{IR} (°)	Sheet to sheet distance (Å)
**1**	2-bromocyclooctanone	160	3:1	Tetrad I	-	8.477
**2**	Nicotine	160	4:1	Tetrad II	-	8.585
**3**	α-thujone	163	4:1 [a]	Tetrad II	-	8.031
**4**	*R*-(+)-limonene	164	4:1 [a]	Tetrad II	-	8.592
**5**	*cis*-rose oxide	175	4:1	Tetrad III	-	8.423
**6**	*cis*-1,2-dimethyl-cyclohexane	135	3:1	DLIC	-	7.926
**7**	1-methoxy-1-methylcyclohexane	143	3:1	DLIC	-	7.827
**8**	2-chlorocyclooctanone	156	3:1	DLIC	-	7.833
**9**	progesterone	332	6:1	DLIC	-	8.837
**10**	sclareolide	292	2:1	“expanded”-DLIC	-	7.717; 15.435
**11**	1,4-dioxane	83	1:1	CLIC	130	7.517
**12**	2-butanol	87	1:1	CLIC	140	7.432
**13**	12-crown-4	170	2:1	CLIC	136	8.830
**14**	γ-terpinene	164	2:1	zz-CLIC I	145	8.260
**15**	eugenol	165	2:1 [a]	zz-CLIC I	130	8.761
**16**	geraniol	185	2:1 [a]	zz-CLIC I	141	8.281
**17**	ROY	218	1:1	“Expanded”-CLIC	152	13.020
**18**	eucalyptol	156	1:1	“Expanded”-CLIC	118	13.649
**19**	15-crown-5	213	1:1	“Disrupted”-CLIC	152	7.905
**20**	guest-free phase	-	-	s-CL	90	7.644

[a] Host:guest ratio was determined by electron density where full guest components could not be modeled.

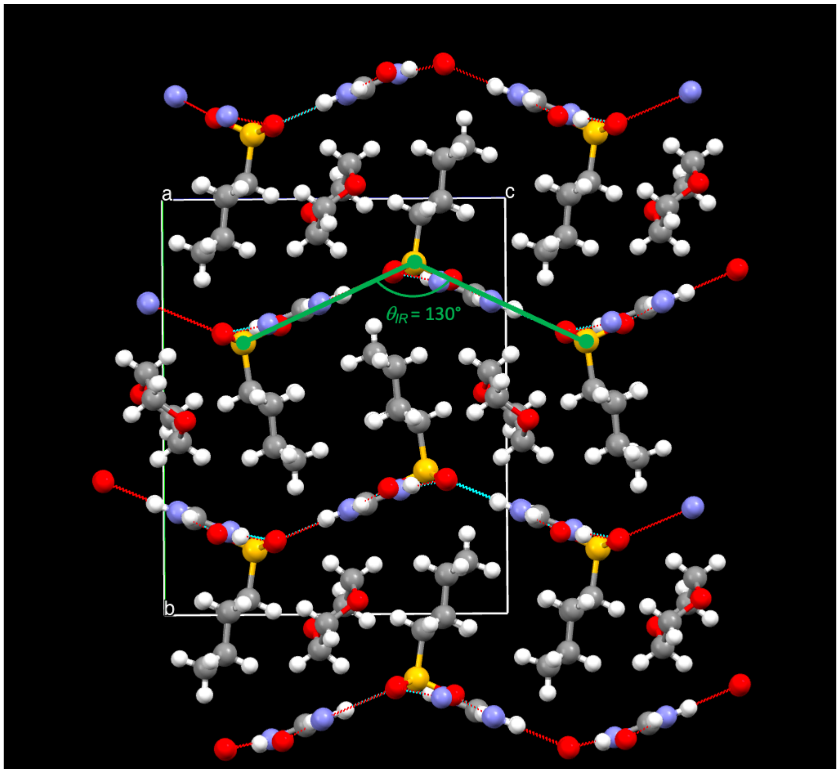

Figure S1. Inter-ribbon puckering angle in (GCHMS)⊃1,4-dioxane (**11**). The term "θ_{R}" corresponds to the inter-ribbon puckering angle. The value of θ_{R} is determined by measuring the angle between three sulfur atoms using a digital protractor, looking down the direction that coincides with the vertex of the two planes that define the puckering. The same protocol was used to determine the puckering angle in compounds forming the CLIC, zz-CLIC and s-CL architectures. Notably, linearity of the hydrogen bonds is preserved in puckered sheets.

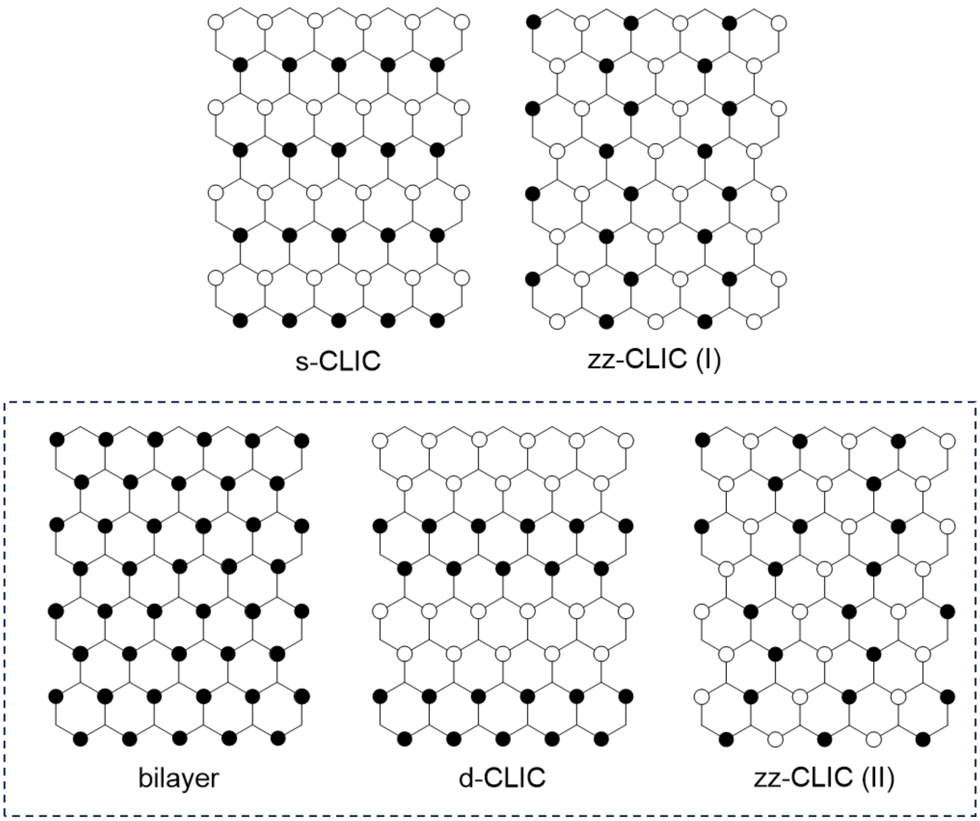

Figure S2. Previously reported projection topologies of guanidinium organomonosulfonate architectures. Filled and open circles depict organic groups projecting from the sulfonate nodes above and below the sheet, respectively. The G ions sit on the undecorated nodes of the hexagonal tiling. The bilayer, d-CLIC, and zz-CLIC (II) architectures have been reported previously for the G monosulfonates but are not observed in the GCHMS compounds described herein.

Table S3. The projection topologies for the GS architectures can be described by a formalism, $M(n)_{d(n)}^{u(n)}m(1)_{d(n)}^{u(n)}m(2)_{d(n)}^{u(n)}$ where $M(n)$, $m(1)$ and $m(2)$ denote n number of major and two minor ribbons, respectively, and u and d are indices that describe the projection sequence of the pillars on the respective ribbons. The number of $M(n)$ terms required for an unambiguous description the projection topology of a given sheet is equal to the number of rows that define a unit translation in the GS sheet along the direction perpendicular to the major ribbon. The major ribbons are chosen, by convention, as those that describe the repeating sequence normal to these ribbons with the least number of $M(n)$ terms. Architectures with identical numbers of up and down pillars on each sheet can be described universally as

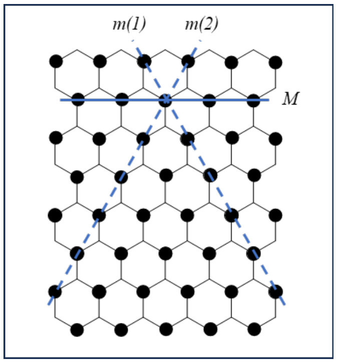

$$M(n)_{d(n)}^{u(n)}m(1)_{d^{i_1},d^{i_2},d^{i_3}\ldots}^{u^{i_1},u^{i_2},u^{i_3}\ldots}m(2)_{d^{i_1},d^{i_2},d^{i_3}\ldots}^{u^{i_1},u^{i_2},u^{i_3}\ldots}$$

where the i, j, k terms need not be identical for the different ribbons but

$$\sum_{k=1,2,3,\ldots}^{u(1)} i_k = \sum_{k=1,2,3,\ldots}^{u(2)} i_k = \sum_n u(n) \quad \text{and}$$

$$\sum_{k=1,2,3,\ldots}^{d(1)} i_k = \sum_{k=1,2,3,\ldots}^{d(2)} i_k = \sum_n d(n).$$

These summation rules are useful because they establish the repeat interval of the projection sequence, which becomes more difficult to assign as the sequence intervals contain more terms in more complex topologies.[8]

Architecture type	Topology formalism	Topology formalism Shorthand
s-CLIC	$M(1)_1^0M(2)_0^1m(1)_1^1m(2)_1^1$	$M_1^0M_0^1$
d-CLIC	$M(1)_{1,1}^{0,0}M(2)_{0,0}^{1,1}m(1)_2^2m(2)_2^2$	$M_{1,1}^{0,0}M_{0,0}^{1,1}$
zz-CLIC I	$M_1^1m(1)_2^2m(2)_2^2$	$M_1^1m(1)_2^2m(2)_2^2$
zz-CLIC II	$M_1^1m(1)_2^2m(2)_3^3$	$M_1^1m(1)_2^2m(2)_3^3$
DLIC	$M(1)_{1,1}^{0,0}M(2)_{0,0}^{1,1}m(1)_2^2m(2)_2^2$	$M_{1,1}^{0,0}M_{0,0}^{1,1}m(1)_2^2m(2)_2^2$
Tetrad III	$M(1)_4^4M(2)_{1,1,2}^{1,2,1}m(1)_4^4m(2)_2^2$	$M_4^4M_{1,1,2}^{1,2,1}m(1)_4^4m(2)_2^2$
Tetrad I	$M_3^3m(1)_{1,2}^{2,1}m(2)_3^3$	$M_3^3m(1)_{1,2}^{2,1}m(2)_3^3$
Tetrad II	$M_3^3m(1)_{1,2}^{2,1}m(2)_3^3$	$M_3^3m(1)_{1,2}^{2,1}m(2)_3^3$

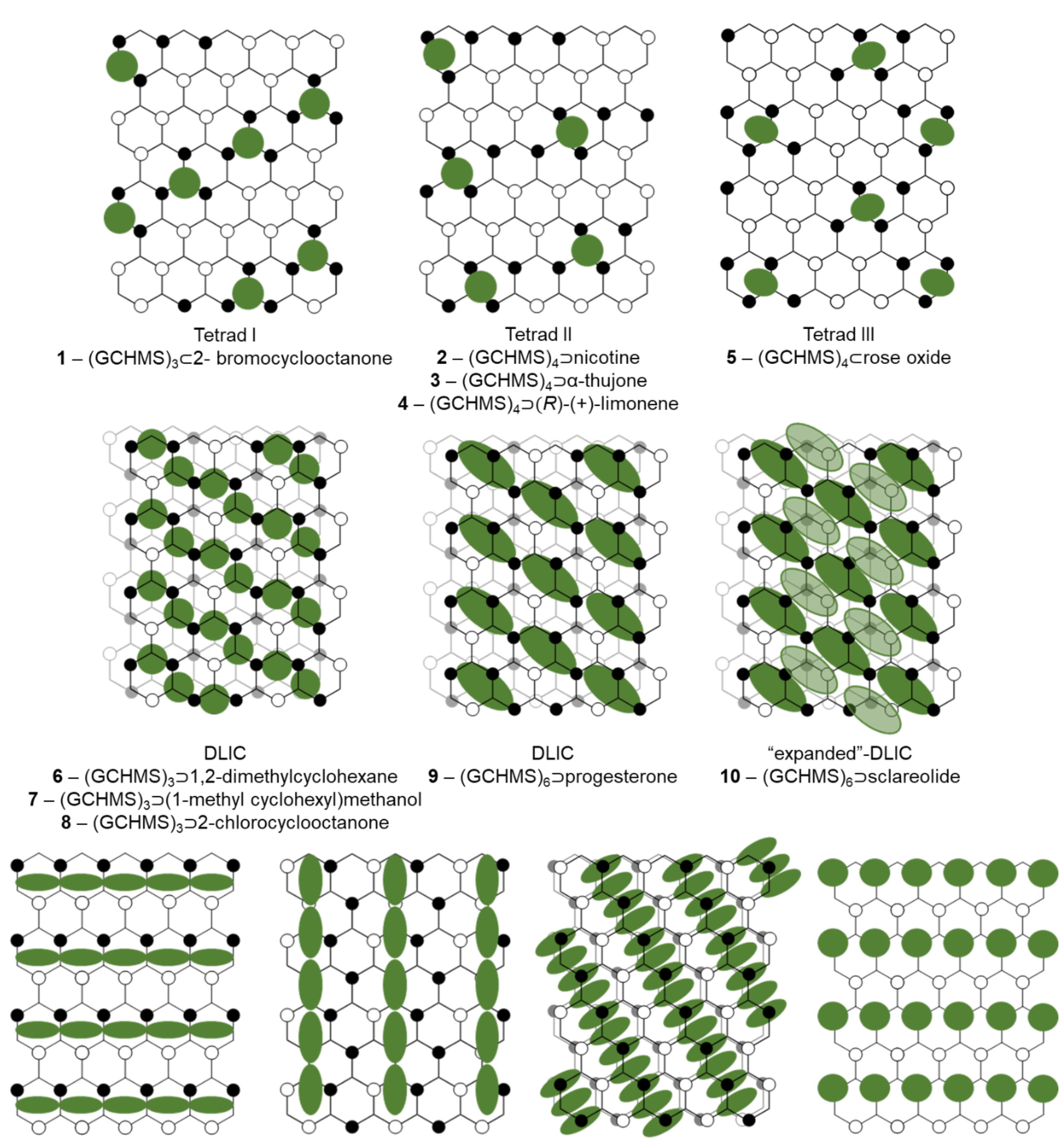

Tetrad I
1 – $(GCHMS)_3\subset$2- bromocyclooctanone

Tetrad II
2 – $(GCHMS)_4\supset$nicotine
3 – $(GCHMS)_4\supset$α-thujone
4 – $(GCHMS)_4\supset$(*R*)-(+)-limonene

Tetrad III
5 – $(GCHMS)_4\subset$rose oxide

DLIC
6 – $(GCHMS)_3\supset$1,2-dimethylcyclohexane
7 – $(GCHMS)_3\supset$(1-methyl cyclohexyl)methanol
8 – $(GCHMS)_3\supset$2-chlorocyclooctanone

DLIC
9 – $(GCHMS)_6\supset$progesterone

“expanded”-DLIC
10 – $(GCHMS)_6\supset$sclareolide

s-CLIC
11 – GCHMS⊃1,4-dioxane
12 – GCHMS⊃2-butanol
13 – $(GCHMS)_2\supset$12-crown-4

zz-CLIC I
14 – $(GCHMS)_2\supset$ γ-terpinene
15 – $(GCHMS)_2\supset$eugenol
16 – $(GCHMS)_2\supset$geraniol

s-CLIC
17 - GCHMS⊂ROY

s-CLIC
18 - GCHMS⊂eucalyptol

Figure S3. Projection topologies of GCHMS architectures. The green circles and ellipsoids symbols denote the guest location as viewed normal to the GS sheets.

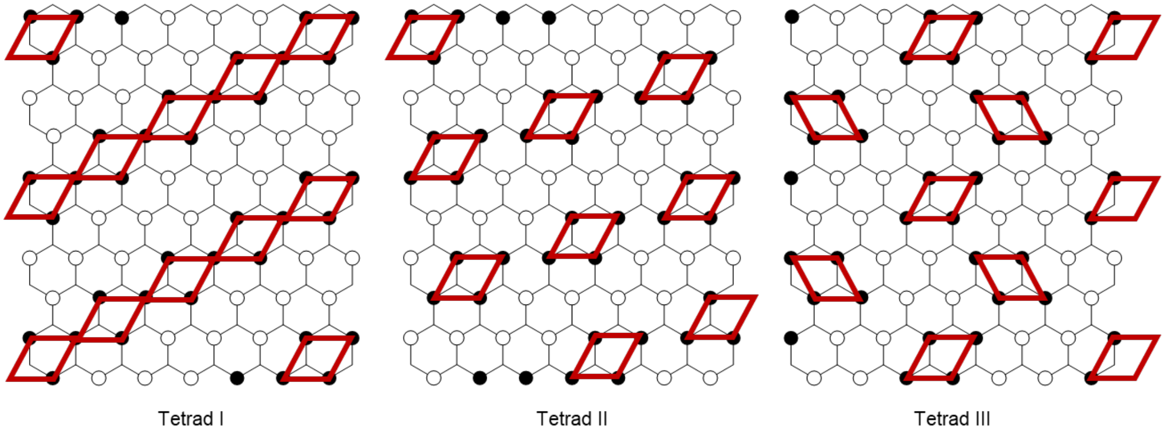

Figure S4. Projection topologies of Tetrad I-III architectures with parallelograms denoting tetrad locations on the GS sheets.

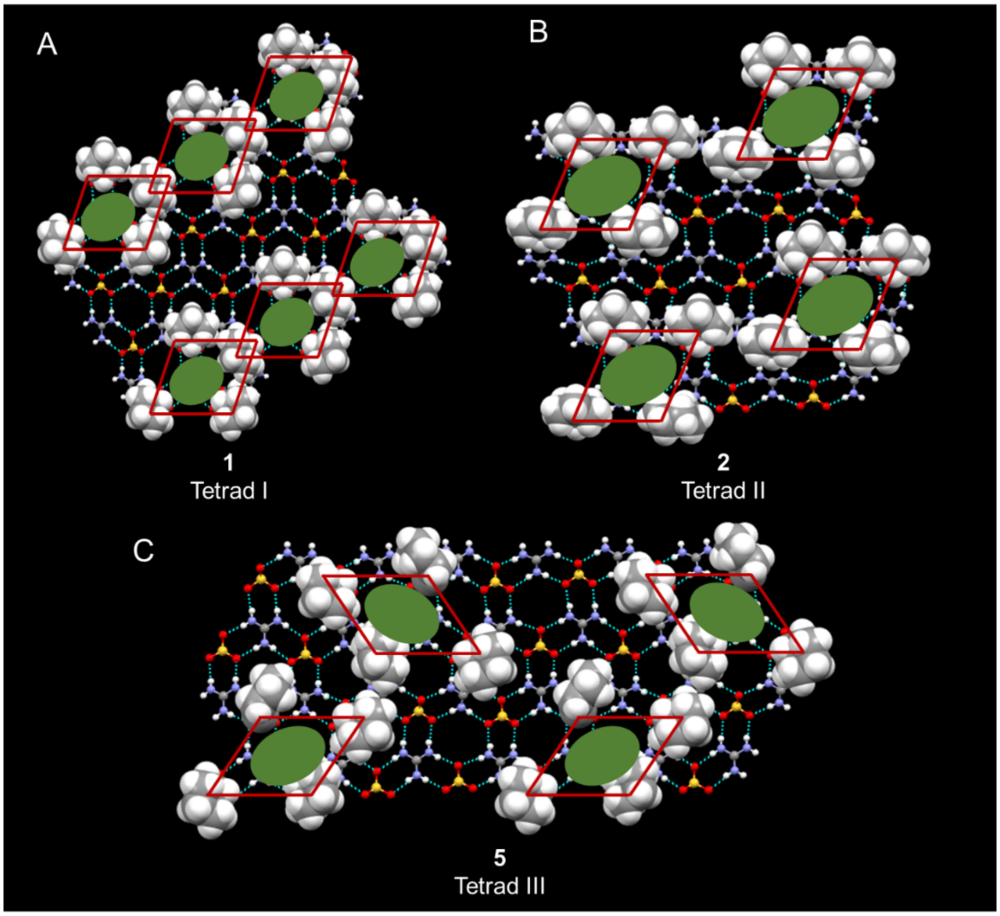

Figure S5. Top-down views of one side of the GS sheet for each Tetrad architecture type described herein, represented here by crystal structures of inclusion compounds (A) **1**, (B) **2**, and (C) **5**. Organic residues are rendered as space-filling. Guest molecules are denoted as green ovals for clarity. Red parallelograms denote the repeat tetrad locations.

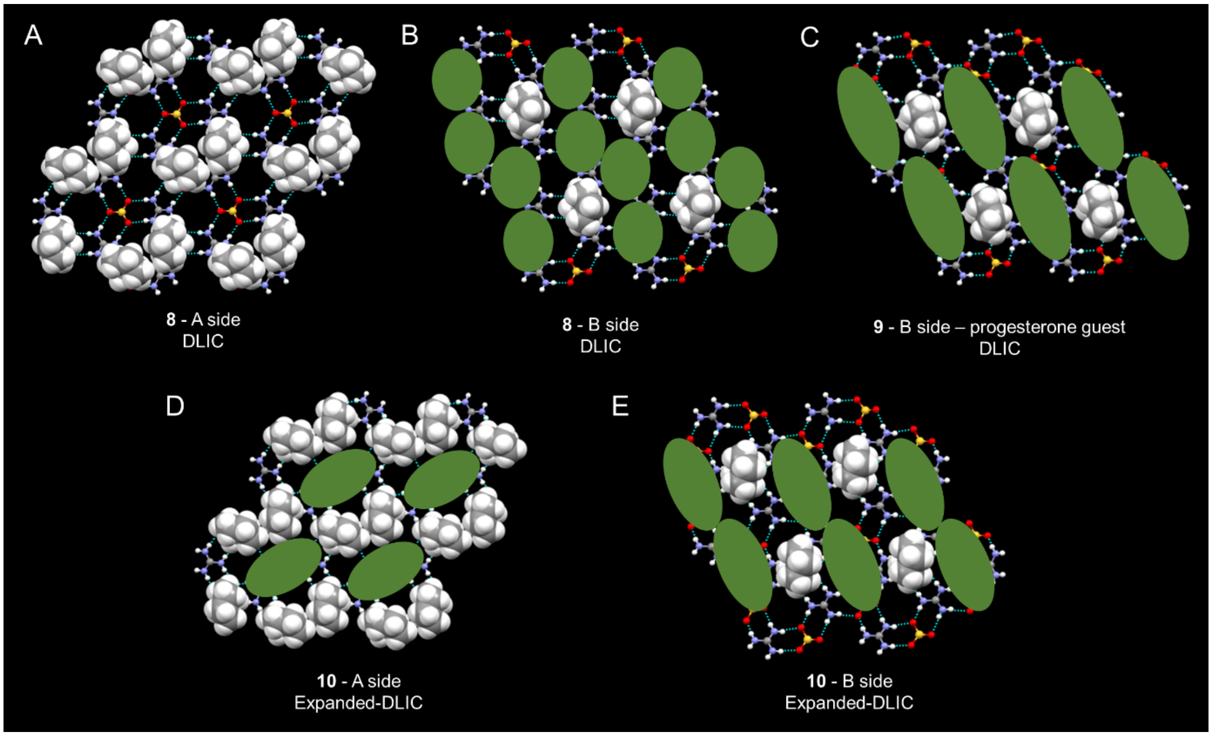

Figure S6. (A-B) Top-down view of the crystal structure of both sides of the GS sheet in **8**, representing the DLIC architecture. (C) Top-down view of the crystal structure of the B side of the GS sheet in **9** showing a larger host:guest ratio in the DLIC architecture. (D-E) Top-down view of the crystal structure of both sides of the GS sheet in **10** in the “expanded”-DLIC architecture. Organic residues are rendered as space-filling. Guest molecules are denoted by green ovals for clarity.

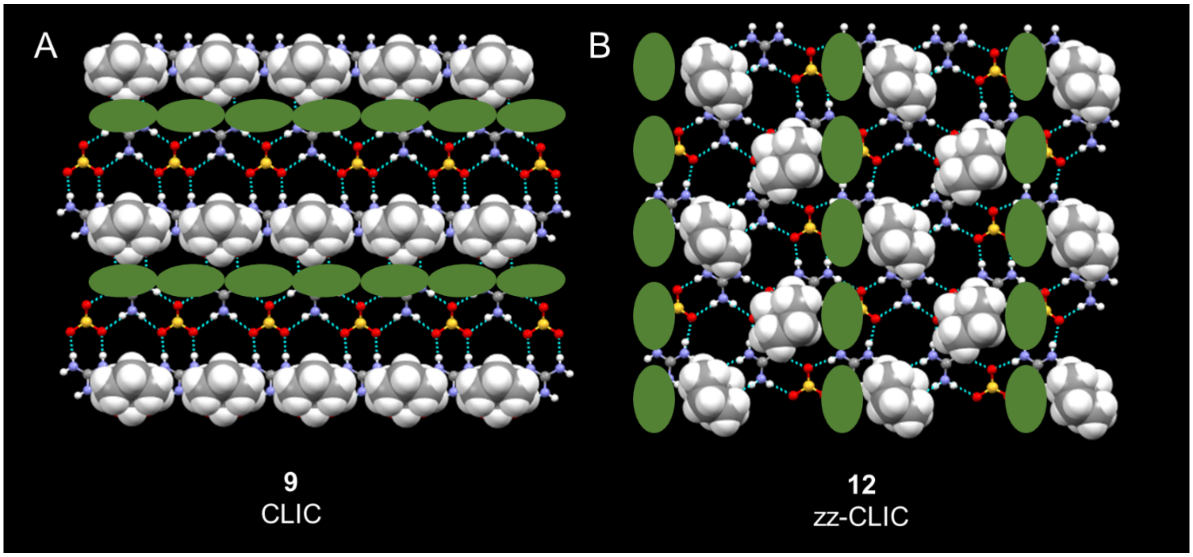

Figure S7. Top-down view of the crystal structures of one side of the GS sheets in (A) **11** and (B) **14**, representing the CLIC and zz-CLIC architectures, respectively. Organic residues are rendered as space-filling. Guest molecules are denoted by green ovals for clarity.

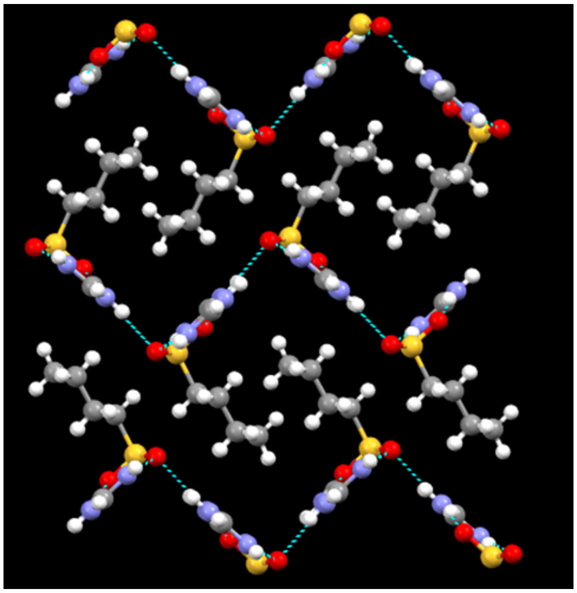

Figure S8. The crystal structure of the guest-free phase of GCHMS (**20**) in the s-CL architecture.

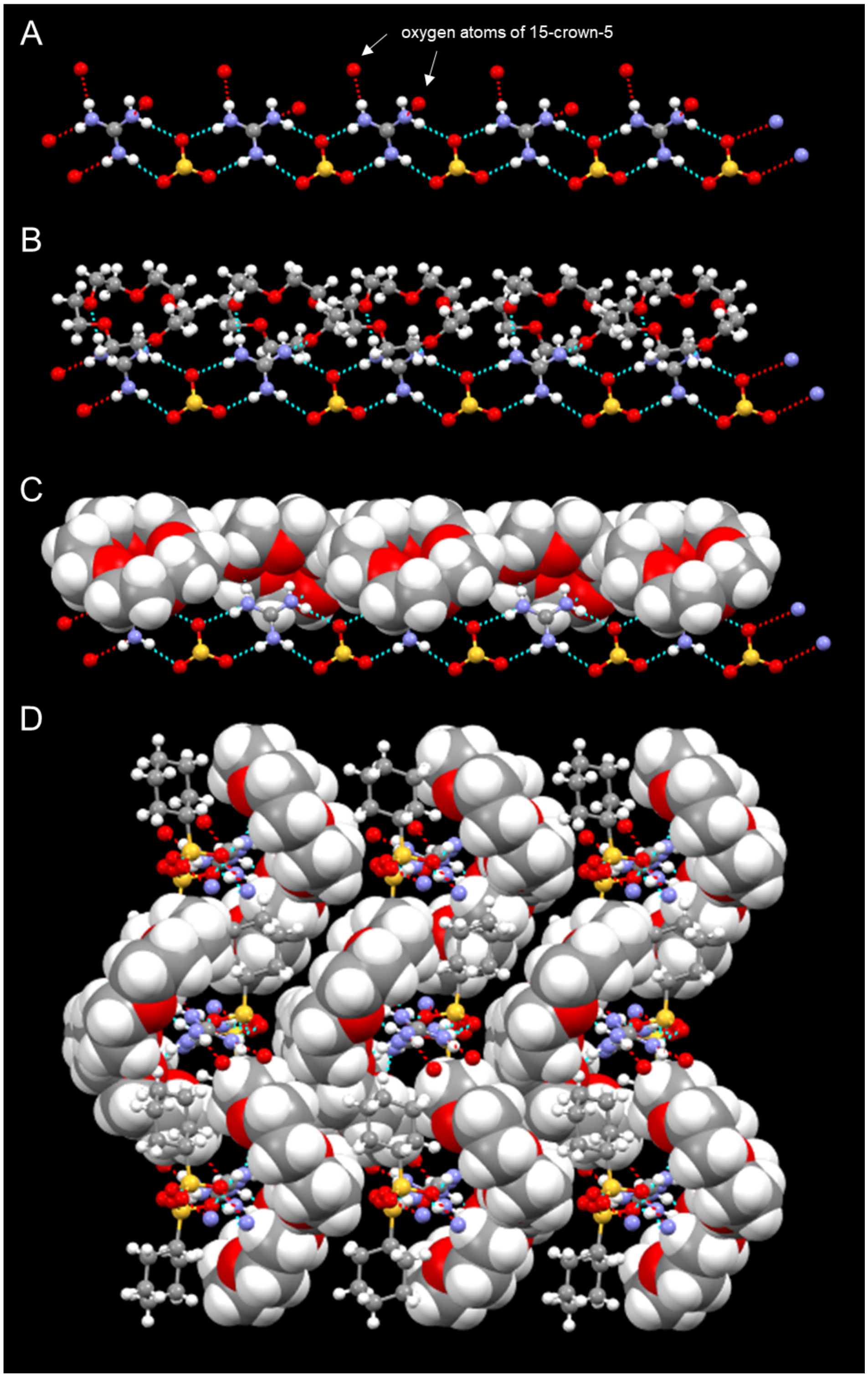

Figure S9. Illustrations of the one-dimensional hydrogen bonded ribbons in GCHMS⊂15-crown-5 (**19**) rendered as (A) ball-and-stick with the CHMS residues and guests removed, (B) as ball-and-stick with guests but CHMS residues removed, and (C) ribbons in ball-and-stick and guests as space-filled. (D) Overall framework as viewed with the hydrogen-bonded ribbons oriented normal to the plane of the page.

References

(1) D. Bartolo, N.; M. Demkiw, K.; M. Valentín, E.; T. Hu, C.; A. Arabi, A.; A. Woerpel, K. Diastereoselective Additions of Allylmagnesium Reagents to α-Substituted Ketones When Stereochemical Models Cannot Be Used. *J. Org. Chem.* **2021**, *86* (10), 7203–7217. https://doi.org/10.1021/acs.joc.1c00553.

(2) APEX4. Bruker AXS: Madison, WI 2020.

(3) Sheldrick, G. M. SHELXT – Integrated Space-Group and Crystal-Structure Determination. *Acta Crystallogr. A* **2015**, *71* (1), 3–8. https://doi.org/10.1107/S2053273314026370.

(4) Sheldrick, G. M. Crystal Structure Refinement with SHELXL. *Acta Crystallogr. C Struct. Chem.* **2015**, *71* (1), 3–8. https://doi.org/10.1107/S2053229614024218.

(5) Dolomanov, O. V.; Bourhis, L. J.; Gildea, R. J.; Howard, J. A. K.; Puschmann, H. OLEX2: A Complete Structure Solution, Refinement and Analysis Program. *J. Appl. Cryst.* **2009**, *42* (2), 339–341. https://doi.org/10.1107/S0021889808042726.

(6) Macrae, C. F.; Sovago, I.; Cottrell, S. J.; Galek, P. T. A.; McCabe, P.; Pidcock, E.; Platings, M.; Shields, G. P.; Stevens, J. S.; Towler, M.; Wood, P. A. Mercury 4.0: From Visualization to Analysis, Design and Prediction. *J. Appl. Crystallogr.* **2020**, *53* (Pt 1), 226–235. https://doi.org/10.1107/S1600576719014092.

(7) Zhao, Y. H.; Abraham, M. H.; Zissimos, A. M. Fast Calculation of van Der Waals Volume as a Sum of Atomic and Bond Contributions and Its Application to Drug Compounds. *J. Org. Chem.* **2003**, *68* (19), 7368–7373. https://doi.org/10.1021/jo034808o.

(8) Holman, K. T.; Martin, S. M.; Parker, D. P.; Ward, M. D. The Generality of Architectural Isomerism in Designer Inclusion Frameworks. *J. Am. Chem. Soc.* **2001**, *123* (19), 4421–4431. https://doi.org/10.1021/ja0030257.
